# Distinct patterns of problematic smartphone use and related factors in Chinese college students

**DOI:** 10.1186/s12888-022-04395-z

**Published:** 2022-11-30

**Authors:** Lan Hong, Xinyi Lai, Dongwu Xu, Wei Zhang, Bichang Wu, Xin Yu, Ke Zhao, Guohua Zhang

**Affiliations:** 1 The Third Hospital of QuZhou, Quzhou, 324000 China; 2 School of Mental Health, Wenzhou Medical University, Wenzhou, 325035 China; 3 Lishui Second People’s Hospital Affiliated to Wenzhou Medical University, Lishui, 323000 China; 4 The Affiliated Kangning Hospital, Wenzhou Medical University, Wenzhou, China; 5 Institute Of Aging, Key Laboratory Of Alzheimer’s Disease Of Zhejiang Province, Wenzhou Medical University, Wenzhou, China

**Keywords:** Problematic smartphone use, Chinese undergraduates, Latent profile analysis, Gender, Depression

## Abstract

**Background:**

This study aimed to categorize different subgroups of problematic smartphone use in Chinese college students. Differences in gender and psychosocial characteristics of the categorized groups were also examined.

**Methods:**

A total of 1123 participants completed the Mobile Phone Addiction Index Scale, the Center for Epidemiologic Studies Depression Scale, the Perceived Social Support Scale, and the Perceived Stress Scale. Using latent profile analysis, we identified different subgroups of problematic smartphone use in college students. Multivariate logistic regression analysis was implemented to examine the relationship between latent classes and demographic and psychosocial covariates.

**Results:**

The four following latent classes were identified: a low-risk group, a moderate-risk with no evasiveness group, a moderate-risk with evasiveness group, and high-risk group that accounted for 11%, 24.1%, 35.5%, and 29.4% of the total sample, respectively. Further analysis revealed that female participants were more likely to be in the moderate-risk with evasiveness and high-risk groups, and individuals with depressed mood were more likely to be in the moderate-risk and high-risk groups.

**Conclusions:**

Classifying college students according to the features of problematic smartphone use is potentially useful for understanding risk factors and developing targeted prevention and intervention programs.

**Supplementary Information:**

The online version contains supplementary material available at 10.1186/s12888-022-04395-z.

## Introduction

Smartphones gained widespread popularity in 2011, and have been increasingly used over the past decade, especially in Mainland China. In August 2021, the China Internet Network Information Center reported that 1.007 billion Chinese people own a personal mobile phone with internet access and that up to 99.6% of them use their smartphone to surf the internet [1]. Because smartphones have permanent access to the internet and can meet a range of demands, users have become extraordinarily attached to these devices. This trend has triggered concern about smartphone overuse among both researchers and members of the general public [2, 3].

One study found that 6.3% of teenagers (6.1% among boys and 6.5% among girls) show signs of problematic smartphone use (PSU) [4], and another reported that the estimated prevalence of PSU in undergraduates in China was 21.3% in 2015 [5]. A review also concluded that the rate of PSU in children and young people was between 10 and 30% from 2011 to 2017 and that the median was close to 25% [6]. Given that excessive smartphone use is a recent phenomenon, research centered on this problem is still emerging, and further empirical studies are needed to support and enrich these critical conversations [3].

Similar to behavioral addictions, excessive smartphone use is associated with a series of adverse effects [7], such as problems related to physical health and cognition (e.g., poor sleep and a decline in self-control) [6, 8], emotional problems (e.g., depression and anxiety) [9, 10], and social issues (e.g., impaired family and school relationships) [11]. Despite these negative effects, the proposal to make mobile phone addiction a new category in the DSM-5 was rejected [12]. Previous literature has viewed smartphones as physical objects akin to “the glass in alcohol addiction” or “the needle in heroin addiction” and thus proposes that we should not ascribe the problems that stem from smartphone usage to the device itself [3]. In other words, the platform and interface of smartphones mean that PSU overlaps with, but is also distinct from, the constructs of addiction [2, 9]. To build on this critical conversation, the current study defined PSU as a maladaptive pattern of mobile phone use, whereby people cannot be separated from their smartphone or control how much they use it, which eventually damages their physical and mental health and hinders their daily functioning [13].

The classification of PSU has differed between previous studies. One method of classification identifies two types of PSU (“yes” and “no”) by setting clear boundaries [14]. The most common approach adopts standard of the mean ± standard deviation (SD), and has mainly divided PSU into three categories: non-addiction group, problematic use group, and addiction group [15]. This variable-oriented method does not reveal different patterns between individuals, however, and may lead to overly generalized conclusions based on the sample [16]. Conversely, a person-oriented method captures information at the individual level and can distinguish meaningful patterns of characteristics in molecular groups [17]. In recent years, person-oriented research methods, such as latent class analysis, have become more popular in the study of disease heterogeneity [18]. Latent profile analysis (LPA) is a form of latent class analysis that is used to assess continuous indicators, and is an empirically derived approach for revealing unobserved heterogeneity in a population by identifying different categories of participants within a given sample [19]. Given that LPA is considered the best method to diagnose class heterogeneity when no clinical interview is available [20, 21], the current study used this approach to explore specific patterns of PSU in college students.

A small body of research has attempted to identify typologies of PSU using latent class analysis [22, 23]. Those studies varied in the number of subgroups that they report, describing anywhere from three to six separate subgroups, and were based on small sample sizes. Potential category characteristics and their influencing factors should thus be explored in more depth using data from a larger sample. Several predictors attributable to high symptom-level subgroups have been identified. Influencing factors such as age [24, 25], low self-esteem [26, 27], loneliness [28], stress [29], affective disorders [30], personality [31], and social relationship [32] have been reported. In this type of research, anxiety and depression have been the main focus [33]. However, these findings are controversial, and a deeper investigation into variability across samples is still needed. College students are a vulnerable group who are easily immersed in their smartphone because they are sensitive to the social surroundings and prone to emotional instability [23]. In summary, the current study performed LPA to identify unobserved and homogeneous subtypes of PSU in college students, and then explored the relationships of PSU types with demographic and psychosocial factors.

## Methods

### Participants and procedures

Data were acquired from a large cross-sectional study. All participants were recruited in December 2018 at Wenzhou Medical University in Wenzhou City, Zhejiang Province, China. The inclusion criteria for the study were as follows: 1) willingness to participate in the baseline measurement and 2) daily smartphone use. Participants were undergraduate students enrolled in a range of medical majors, including psychiatry, clinical medicine, and Traditional Chinese Medicine. A total of 1150 subjects completed the survey anonymously. Twenty-seven (2.3%) were excluded because more than 20% of the data was missing on one or more scales of key psychological variables. Thus, the final study sample consisted of 1123 (97.7%) participants.

### Measures

#### Self-reported demographic survey

While under the supervision of trained evaluators, participants completed the social and demographic survey. This collected general information from participants, such as gender, grade, residence, and single-child status.

#### Mobile phone addiction index scale

The Mobile Phone Addiction Index Scale was used to quantitatively assess participants’ mobile phone use levels [34]. The scale assesses four dimensions, including inability to control cravings, feeling anxious and lost, withdrawal or escape, and loss of productivity [35]. The scale includes 17 items, which are rated on a 5-point Likert scale. The higher the score, the stronger the PSU. The Cronbach’s alpha for the scale was 0.82 in the present study.

#### Depression scale

The Center for Epidemiologic Studies Depression Scale [36] was used to assess depression status. The scale comprises 20 items, each of which are scored according to the severity of symptoms, and which assess nine symptoms of depression [37]. A higher score indicates more severe symptoms of depression. The Center for Epidemiologic Studies Depression Scale is one of the most widely used self-report scales because it has good psychometric properties that allow for the assessment of depressive symptoms in the general public [36]. The Cronbach’s alpha for this scale was 0.86 in the present study.

#### Perceived social support scale

The 12-item Perceived Social Support Scale was used to assess the levels of perceived social support [38, 39]. The scale assesses three dimensions: family support, friend support, and other forms of support (e.g., teachers and relatives). The scale has good reliability and validity in Chinese populations [40]. A higher score indicates more available social support. The Cronbach’s α for the scale was 0.86 in the present study.

#### Perceived stress scale

The Perceived Stress Scale [41] was used to measure the extent to which respondents felt that their stress was unpredictable, uncontrollable, and overwhelming. The scale consists of 14 items scored on a 5-point Likert scale ranging from never (0) to frequently (4). Total scores ranged from 0 to 56, with higher scores indicating greater perceived stress. The Cronbach’s α for the scale in the current sample was 0.73.

### Statistical analysis

#### Identification of potential categories

M-plus version 8.0 [42] was used to construct an LPA so that any heterogeneous latent category differences in PSU could be characterized. LPA provides classification of individuals and is a special form of finite mixture modeling. Unlike other approaches, such as cluster analysis, cases are not absolutely assigned to a class, but have a certain probability of belonging to a class [43]. This probability-based and individual-centered approach can reduce misclassification and missing rates of participants [44].

The evaluation indicators of the degree of fit of an LPA are the Akaike Information Criterion (AIC) [45], Bayesian Information Criterion (BIC) [46], and sample-size adjusted BIC (aBIC) [47]. This is a relative metric, whereby lower BIC, AIC, and aBIC values indicate a better model fit [47]. The fourth indicator is the entropy value, with a maximum value of 1 and high values preferred [48]. An entropy value greater than 0.8 indicates a classification accuracy of over 90% [49]. Priority was given to entropy in cases where fit indices between the two models were relatively similar. The bootstrapped likelihood ratio test and Lo–Mendell–Rubin test were also applied. A significant likelihood ratio test for k classes with *p* < 0.05 indicates that the specified k-class model is an improvement over a model with k-1 classes [50]. To avoid solutions based on local maxima, we used 200 random sets of starting values initially and 50 final stage optimizations. Additionally, each latent class was defined with meaningful clinical interpretability [51]. Posterior probabilities from the model were used to assign each participant to their most likely class [18].

#### Identification of risk factors

Rather than deleting missing values, we replaced them with average values. All categorical variables are described as counts and percentages, and all continuous variables are described as means and SDs. Using the classification results of the potential categories as dependent variables and the demographic factors, rates of depression, perceived social support, and perceived stress scores as independent variables, a multinomial logistic regression model was established using SPSS version 22.0. Odds ratios (ORs) with 95% confidence intervals (CIs) are reported with a significance level set at 5%.

## Results

### Participant characteristics

A total of 1123 college students participated in this study. Descriptive statistics for the sample are presented in Table 1. A total of 704 participants (62.6%) were female, and 419 (37.4%) were male. The majority lived in the city (*n* = 636, 56.6%), and 592 (52.7%) were not an only child. The overall mean Center for Epidemiologic Studies Depression Scale score was 36 (SD = 9.3).

**Table 1:** Demographic characteristics of the sample

Characteristics	Total (*n* = 1123)			
	n	%	Mean	SD
Gender				
Female	704	62.6		
Male	419	37.4		
Grade				
Freshman	346	30.8		
Sophomore	414	36.9		
Junior	363	32.3		
Family Origin				
City	636	56.6		
Rural	487	43.4		
One-child family				
Yes	531	47.3		
No	592	52.7		
CES-D			36.0	9.3
PSSS			61.5	14.4
PSS			39.3	6.4
MPAI			48.5	10.8

*SD* standard deviation, *CES-D* Center for Epidemiologic Studies Depression Scale, *PSSS* Perceived Social Support Scale, *PSS* Perceived Stress Scale, *MPAI* Mobile Phone Addiction Index Scale

### Latent profile analysis

Using a person-centered approach, LPA was used to identify PSU in college students. Five latent class models were estimated, and the fit indices of the generated models are reported in Table 2. The AIC, BIC, and aBIC continuously decreased as the number of latent classes increased. The Lo-Mendell-Rubin test value of the five class solutions was not significant (*p* >  0.05). Compared with the two- and three-class solutions, the AIC, BIC, and aBIC values indicated that the four-class solution was preferable, as did the entropy value. The high posterior probabilities of memberships of the four latent classes (0.943, 0.889, 0.914, and 0.936, respectively) also indicated good discrimination of the model. The four-class solution was thus selected as the optimal solution.

**Table 2:** Model fit indices for one- to five-profile pattern of MPAI items and profile prevalence (%) of LPA (*n* = 1123)

No. of classes	AIC	BIC	aBIC	Entropy	LMR	BLRT	Proportion of individuals in Category
1	60,560.119	60,730.927	60,622.933	–	–	–	–
2	58,054.385	58,315.621	58,150.454	0.812	< 0.001	< 0.001	47.1/52.9
3	57,288.773	57,640.436	57,418.097	0.843	< 0.05	< 0.001	12.2/55.7/32.0
**4**	**56,707.082**	**57,149.173**	**56,869.661**	**0.849**	**< 0.05**	**< 0.001**	**11.0/24.1/35.5/29.4**
5	56,397.232	56,929.750	56,593.065	0.821	> 0.05	< 0.001	10.4/22.5/23.3/20.0/23.7

The values reported in this table are hypothetically derived for illustrative purposes. *AIC* Akaike information criterion; *BIC* Bayesian information criterion; *aBIC* adjust Bayesian information criterion; *BLRT* bootstrap likelihood ratio test; *LMR* Lo-Mendell-Rubin test; Bold indicates the selected category

**Fig. 1:**
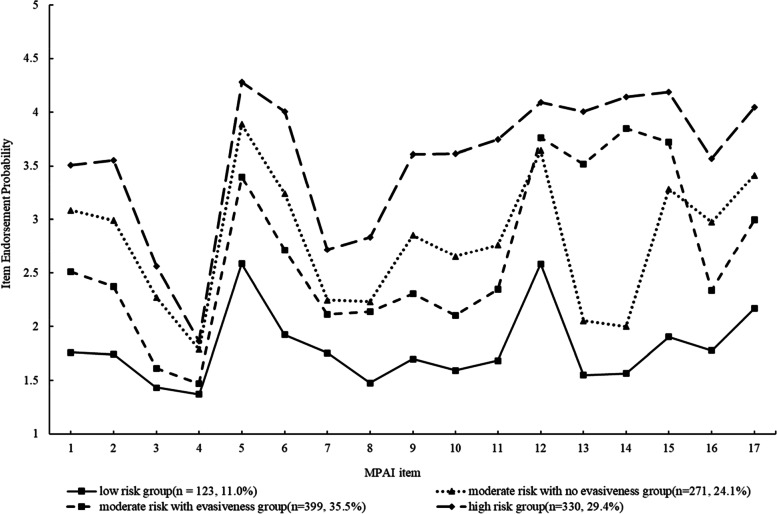
Profiles for 4-class LPA model of PSU

Our results revealed a four classes solution that was hierarchically organized, meaning that the classes varied from the highest to the lowest frequencies of symptom endorsement. Figure 1 depicts the profiles of PSU across the four classes. Class 1 was termed the “low-risk group” (*n* = 123, 11%) due to the fact that this subgroup had the lowest Mobile Phone Addiction Index Scale scores. Classes 2 and 3 demonstrated more severe smartphone-related problems than this low-risk group. Class 3 scored significantly higher than Class 2 on evasiveness (item 13: “When I feel isolated, I use my phone to chat with others”; item 14: “When I feel lonely, I use my phone to chat with others”; item 15: “When the mood is low, I play with my phone to improve my mood”). Thus, Class 3 was named the “moderate-risk with evasiveness group” (*n* = 399, 35.5%) and Class 2 was called the “moderate-risk with no evasiveness group” (*n* = 271, 24.1%). Class 4 comprised approximately 29.4% of the sample (*n* = 330). Participants in this group showed a poorer psychosocial profile with respect to their inability to control cravings, feeling anxious and lost, withdrawal or escape, and productivity loss. Individuals in Class 4 were the most likely to experience all the different forms of PSU. This class was labeled the “high-risk group”.

### Predicting class membership

Using the “low-risk group” as the reference class, the multinomial regression analysis results are shown in Table 3. There were significant sex-related differences between classes. Female participants were more likely to exhibit PSU than male participants. Female participants were 1.96 (95% CI: 1.27–3.00) and 2.23 times (95% CI: 1.43–3.46) more likely to belong to the moderate-risk with evasiveness group and high-risk group than were male participants. Additionally, depression increased the odds of participants falling into Class 2 (OR = 1.08, 95% CI: 1.05–1.11), Class 3 (OR = 1.04, 95% CI: 1.02–1.07), or Class 4 (OR = 1.11, 95% CI: 1.08–1.15).

**Table 3:** Predictors of the latent group membership for the PSU based on the multinomial regression

Variables	Moderate risk with no evasiveness group (*n* = 271, 24.1%)			Moderate risk with evasiveness group (*n* = 399, 35.5%)			High risk group (*n* = 330, 29.4%)		
	OR	95% CI	*p*	OR	95% CI	*p*	OR	95% CI	*p*
Gender Female Male	1.24 ref	0.79–1.94	0.347	1.96	1.27–3.00	0.002	2.23	1.43–3.46	< .001
Grade Freshman Sophomore Junior	0.78 0.72 ref	0.44–1.37 0.43–1.20	0.383 0.206	0.85 0.81	0.49–1.46 0.50–1.34	0.553 0.206	0.85 0.63	0.49–1.47 0.38–1.05	0.551 0.076
Family Origin Rural City	0.96 ref	0.60–1.52	0.844	0.75	0.48–1.17	0.205	0.89	0.56–1.40	0.603
Single child Yes No	1.06 ref	0.66–1.69	0.817	1.49	0.95–2.33	0.084	1.28	0.81–2.04	0.288
CES-D	1.08	1.05–1.11	< .001	1.04	1.02–1.07	0.003	1.11	1.08–1.15	< .001
PSSS	0.98	0.96–1.02	0.50	0.99	0.97–1.03	0.83	0.98	0.95–1.02	0.400
PSS	1.01	0.93–1.09	0.85	1.02	0.94–1.09	0.68	0.99	0.91–1.08	0.820

The reference category is “low risk group”. *CI* confidence interval; *OR* odds ratio; *CES-D* Center for Epidemiologic Studies Depression Scale; *PSSS* Perceived Social Support Scale; *PSS* Perceived Stress Scale

## Discussion

The present study aimed to identify different types of PSU in Chinese college students and to assess factors associated with the different types of PSU. According to the present results, we make recommendations for future prevention and intervention methods. Past research on PSU has demonstrated a heterogeneous and hierarchical organization by identifying classes of individuals based on their symptoms [22, 23]. Results from the LPA in our study supported a four-class model sorted by frequency of symptoms, as follows: (a) the low-risk group, (b) the moderate-risk with no evasiveness group, (c) the moderate-risk with evasiveness group, and (d) the high-risk group. This classification supported the hierarchical nature of PSU in college students and revealed differences in PSU types.

The low-risk group comprised adolescents that displayed low frequencies on all PSU symptoms. This subgroup included the least participants (*n* = 123, 11.0%). The high-risk group class included 29.4% (*n* = 330) of all participants. Although most studies [22, 52] have reported larger low-risk groups than high-risk groups, our findings are not consistent with this. However, it is important to consider that belonging to the high-risk group does not necessarily mean that the participants showed clinically relevant signs of PSU. Indeed, they only scored significantly higher in the Mobile Phone Addiction Questionnaire. Discrepancies compared to previous studies might also be due to the different instruments used and/or differences among participants. Additionally, previous research has concluded that Internet addiction is associated with rapid national development. This is because a country’s development is tied to advances in new media and technology in many aspects of life, which can lead to excessive Internet use [53]. Similarly, our study reveals the diversity of PSU and shows how its prevalence varies across regions.

Most participants belonged to the moderate-risk group (*n* = 670, 59.6%) of all the four classes. This is consistent with previous results [31, 54]. The moderate-risk group was divided into two groups, namely, groups with and without evasiveness. Class 3 scored higher on the evasiveness dimension compared to Class 2, which is a result rarely seen in previous studies. One study have shown that PSU and loneliness are significantly and positively correlated, and that loneliness is a major predictor of addiction to social networking services [55]. Similarly, PSU has been associated with trait anxiety [9, 30]. Based on these findings and the observation that Class 3 scored higher on items related to emotion regulation, we can speculate that Class 3 used smartphones more frequently to regulate negative mood, which is a sign of behavioral addiction. We therefore suggest that Class 3 has a slightly higher risk of PSU (or addiction) than Class 2.

According to our results of multinomial logistic regression analyses, female college students were more likely to belong to the high-risk group. A previous study showed that the overuse of mobile phones was more common in girls than in boys [56]. Moreover, female participants show higher levels of attachment to and dependence on smartphones [57, 58], and women are more likely to be classified as being high-risk for mobile phone addiction [24]. Surveys have shown that women aged 20 or older are three times more likely than men (25% vs. 9%) to agree with the statement, “I can’t imagine life without my phone” [59]. Our research supports the “social factor” hypothesis, which argues that women are more vulnerable than men in social environments [60]. This may mean that women are more susceptible to PSU in the campus environment compared to men.

Depression severity was significantly associated with PSU, which is consistent with previous findings [9]. Recent research has shown that PSU severity was moderately correlated with anxiety and depression severity [61], and that this association extends to adults of all ages [62]. PSU and depression interrelationships found in prospective cohort studies are likely to be significantly bi-directional [63, 64]. On the one hand, PSU is associated with a lack of social support, which can induce emotional disorders such as anxiety and depression [65]. Smartphone overuse and tolerance could cause people to use their smartphones for long periods of time at night, which can lead to sleep problems that could lead to anxiety and depression [66]. On the other hand, PSU has an impact on neural activity by affecting rewording progress [67]. Phone use usually offers rewards to people and therefore ensures that the behavior will reoccur [68]. As a result of this newly established reward mechanism, when an individual puts down their mobile phone and returns to daily life, satisfaction is not as easy or quick to attain, which could lead to dissatisfaction and depression. Additionally, individuals with depressive moods are more vulnerable to PSU. Compensatory Internet use theory suggests that people with negative emotions may alleviate their bad moods through excessive smartphone use, which suggests that people with depression are more susceptible to PSU than psychologically healthy people [69]. Recent research has considered PSU as a coping mechanism to eliminate negative emotions, trigger positive feelings, and compensate for a lack of offline socialization [70]. This compensation for negative emotions was also reflected in our study.

Perceived social support and perceived stress were not significantly different between the four latent groups, and were not identified as important influencing factors of PSU in our research. Recent work has suggested that real-life social support helps to reduce PSU [69], but other studies have observed no such association [11, 71]. According to a review on adolescents’ PSU, whether social networks can be considered as a predictive factor remains open to debate [12]. Combined with the current results, we have reason to doubt that the effect from perceived social support was weakened by depression. The results of one study confirmed that the direct effect of social support on mobile phone addiction only accounted for 12% of the total effect, and their association was mediated by depression [72]. This supports the compensatory internet use theory, whereby college students who lack sufficient social support are more likely to experience a depressed mood, and more inclined to overuse their smartphone for comfort and relationships [69]. Although perceived stress was found to be a predictor of PSU in a previous study [5], our contrasting result supports the Interaction of Person-Affect-Cognition-Execution model proposed by Brand et al. [73]. This process model posits that individuals with Internet use disorder are more inclined to display problematic behavior to regulate emotions, possibly due to the interaction of more vulnerable traits when facing stress and inappropriate coping strategies when confronted with stressful situations. In other words, the association between perceived stress and PSU can present an indirect pathway, such as a mediating effect [29]. Therefore, based on our findings, future work should further explore the mechanisms underlying the relationship between perceived stress and PSU in college students.

Our findings indicate that timely and effective psychological interventions could help reduce PSU in college students. First of all, universities and their psychological providers should develop targeted educational programs and guidelines for students [74], with relevant guidelines and courses offered for different genders. Students in moderate and high-risk PSU groups should be helped to enhance their adaptive coping skills and focus on real life instead of smartphones [5]. Moreover, researchers have advocated improving emotional management and fostering emotion intelligence [2]. Finally, teachers and psychological providers should pay full attention to psychological interventions and treatment of students with depression symptoms and establish mental health files for high-risk students.

## Limitations

This study has several limitations. First, the data were collected from only one university, which may limit the generalizability of these findings. Future research should examine PSU in a sample that is more representative of the general population. Second, the participants were all medical students who did not exhibit significant functional impairments. Future studies should expand to the clinical setting, and compare their findings with those of this study. Third, this was a cross-sectional study, which cannot infer causal relationship of college students’ PSU. Furthermore, the use of neurocognitive tests or neurobiological markers would increase the validity of the results [31]. Regarding the methodology, the characteristics of LPA are divided according to relative probability. Future research could try to identify more discriminative grouping and explore the consistency of scale cut-off value division and LPA grouping after fully considering the above limitations.

## Conclusion

This study identified four trajectories of PSU and the factors associated with each. The results demonstrated that being female and exhibiting symptoms of depression are risk factors for PSU, yet depressed mood might be a negative consequence of PSU. As an extension of this study, it might be possible to achieve early identification of college students at high risk of PSU. To improve PSU, more attention should be paid to individuals with risk factors, female college students and students with depression.

## Supplementary Information

**Additional file 1.**

**Additional file 2.**

## Data Availability

The data is available on request from the Department of Psychology, Wenzhou Medical University (see Additional file 1). Guohua Zhang had received permission and accessed to all the data.

## Abbreviations

PSU: Problematic smartphone use
LCA: Latent class analysis
LPA: Latent profile analysis
BIC: Bayesian information criterion
aBIC: Adjust Bayesian information criterion
BLRT: Bootstrap likelihood ratio test
LMR: Lo-Mendell-Rubin test
CI: Confidence interval
OR: Odds ratio
CES-D: Center for Epidemiologic Studies Depression Scale
PSSS: Perceived Social Support Scale
PSS: Perceived Stress Scale

## References

[1] The 48 Statistical Report on InternetDevelopment in China [http://www.china.org.cn/china/InternetReports/node_1241550.htm].

[2] Busch PA, Mccarthy S (2021). Antecedents and consequences of problematic smartphone use: a systematic literature review of an emerging research area. Comput Hum Behav.

[3] Panova T, Carbonell X (2018). Is smartphone addiction really an addiction?. J Behav Addict.

[4] Méndez I, Jorquera Hernández AB, Ruiz-Esteban C (2020). Profiles of Mobile phone problem use in bullying and cyberbullying among adolescents. Front Psychol.

[5] Long J, Liu TQ, Liao YH, Qi C, He HY, Chen SB, Billieux J (2016). Prevalence and correlates of problematic smartphone use in a large random sample of Chinese undergraduates. Bmc Psychiatry.

[6] Sohn SY, Rees P, Wildridge B, Kalk NJ, Carter B (2019). Prevalence of problematic smartphone usage and associated mental health outcomes amongst children and young people: a systematic review, meta-analysis and GRADE of the evidence. BMC psychiatry.

[7] Derevensky JL, Hayman V, Gilbeau L (2019). Behavioral addictions: excessive gambling, gaming, internet, and smartphone use among children and adolescents. Pediatr Clin N Am.

[8] Velthoven MV, Powell J, Powell G (2018). Problematic smartphone use: digital approaches to an emerging public health problem. Digital Health.

[9] Elhai JD, Levine JC, Dvorak RD, Hall BJ (2017). Problematic smartphone use: a conceptual overview and systematic review of relations with anxiety and depression psychopathology. J Affect Disord.

[10] Sahu M, Gandhi S, Sharma MK (2019). Mobile phone addiction among children and adolescents: a systematic review. J Addict Nurs.

[11] Roser K, Schoeni A, Foerster M, Rsli M (2016). Problematic mobile phone use of Swiss adolescents: is it linked with mental health or behaviour?. Int J Public Health.

[12] Fischer-Grote L, Kothgassner OD, Felnhofer A (2019). Risk factors for problematic smartphone use in children and adolescents: areview of existing literature. Springer Open Choice.

[13] Wang X (2012). On the Relationship Between College Students' Mobile Phone Addiction,Loneliness and Personality (in Chinese). Chin J Spec Educ.

[14] Wang HH, Wang MC, Wu SG (2015). Mobile phone addiction symptom profiles related to interpersonal relationship and loneliness for college students: a latent profile analysis. Chin J Clin Psychol.

[15] Eye AV, Bergman LR (2003). Research strategies in developmental psychopathology: dimensional identity and the person-oriented approach. Dev Psychopathol.

[16] Wanders RB, Loo H, Vermunt JK, Meijer RR, Jonge PD (2016). Casting wider nets for anxiety and depression: disability-driven cross-diagnostic subtypes in a large cohort. Psychol Med.

[17] Ling Y, Liu C, E. SH, Zeng Y, Zhao N, Li Z: A study on classification features of depressive symptoms in adolescents. J Mental Health (Abingdon, England) 2021, 30(2):208–215. 10.1080/09638237.2019.1677865 31656127

[18] Lubke GH, Muthén B (2005). Investigating population heterogeneity with factor mixture models. Psychol Methods.

[19] Li JB, Wu A, Feng LF, Deng Y, Li JH, Chen YX, Mai JC, Mo P, Lau J (2020). Classification of probable online social networking addiction: a latent profile analysis from a large-scale survey among Chinese adolescents. J Behav Addict.

[20] Carr MM, Grilo CM (2020). Examining heterogeneity of binge-eating disorder using latent class analysis. J Psychiatr Res.

[21] Elhai JD, Rozgonjuk D, Yildirim C, Alghraibeh AM, Alafnan AA (2019). Worry and anger are associated with latent classes of problematic smartphone use severity among college students. J Affect Disord.

[22] Yue H, Zhang X, Sun J, Liu M, Li C, Bao H (2021). The relationships between negative emotions and latent classes of smartphone addiction. PLoS One.

[23] Hussain Z, Griffiths MD, Sheffield D (2017). An investigation into problematic smartphone use: the role of narcissism, anxiety, and personality factors. J Behav Addict.

[24] Walsh SP, White KM, Cox S, Young RM (2011). Keeping in constant touch: the predictors of young Australians' mobile phone involvement. Comput Hum Behav.

[25] Kim E, Koh E (2018). Avoidant attachment and smartphone addiction in college students: the mediating effects of anxiety and self-esteem. Comput Hum Behav.

[26] You Z, Zhang Y, Zhang L, Xu Y, Chen X (2019). How does self-esteem affect mobile phone addiction? The mediating role of social anxiety and interpersonal sensitivity. Psychiatry Res.

[27] Bian M, Leung L (2015). Linking loneliness, shyness, smartphone addiction symptoms, and patterns of smartphone use to social capital. Soc Sci Comput Rev.

[28] Yang X, Wang P, Hu P (2020). Trait procrastination and Mobile phone addiction among Chinese college students: a moderated mediation model of stress and gender. Front Psychol.

[29] Jocelyne MB, Doris J, Naoyuki H (2017). Depression, anxiety, and smartphone addiction in university students- a cross sectional study. PLoS One.

[30] Mok JY, Choi SW, Kim DJ, Choi JS, Song WY (2014). Latent class analysis on internet and smartphone addiction in college students. Neuropsychiatric Dis Treatment.

[31] Salehan M, Negahban A (2013). Social networking on smartphones: when mobile phones become addictive. Comput Hum Behav.

[32] De-Sola Gutiérrez J (2016). Rodríguez de Fonseca F, Rubio G: cell-phone addiction: a review. Front Psychiatr.

[33] Huang H, Niu LY, Zhou CY, Ming WH (2014). Reliability and validity of Mobile phone addiction index for Chinese college students (in Chinese). Chin J Clin Psychol.

[34] Leung L (2008). Linking psychological attributes to addiction and improper use of the mobile phone among adolescents in Hong Kong. J Children Media.

[35] Radloff LS (1977). The CES-D scale a self-report depression scale for research in the general population. Appl Psychol Meas.

[36] Li J, Zhao F, Bai H, Lin P, Shao D, Sun J, Zhang H, Cao F (2019). Psychometric properties of the Chinese version of the Center for Epidemiologic Studies Depression Scale-Revised in patients with cancer: a cross-sectional study. Int J Nurs Stud.

[37] Dahlem NW, Zimet GD, Walker RR (1991). The multidimensional scale of perceived social support: a confirmation study. J Clin Psychol.

[38] Yan BB, Zheng X (2006). Researches into relations among social-support, self-esteem and subjective well-being of college students. Psychol Dev Education.

[39] Huang Y, Wu R, Wu J, Yang Q, Zheng S, Wu K (2020). Psychological resilience, self-acceptance, perceived social support and their associations with mental health of incarcerated offenders in China. Asian J Psychiatr.

[40] Cohen S, Kamarck T, Mermelstein R (1983). A global measure of perceived stress. J Health Soc Behav.

[41] Muthén LK (1998). Muthén BO: Mplus User’s Guide.

[42] Muthén L, Muthén B (1998). Mplus user's guide.

[43] Magidson J, Vermunt JK (2022). Latent class models for clustering: a comparison with K-means. Canad J Market Res.

[44] Akaike H (1987). Factor analysis and AIC. Psychometrika.

[45] Schwarz GE (1978). Estimating the dimension of a model. Ann Stat.

[46] Sclove SL (1987). Application of model-selection criteria to some problems in multivariate analysis. Psychometrika.

[47] Carragher N, Adamson G, Bunting B, McCann S (2009). Subtypes of depression in a nationally representative sample. J Affect Disord.

[48] Lubke GH, Muthén BO (2007). Performance of factor mixture models as a function of model size, covariate effects, and class-specific parameters. Structural Equation Modeling A Multidisciplinary Journal.

[49] Muthén L, Muthén B. Mplus user's guide (version 7.2). Los Angeles, CA; 2012.

[50] Nylund KL, Asparouhov T, Muthén BO (2007). Deciding on the number of classes in latent class analysis and growth mixture modeling: a Monte Carlo simulation study. Structural Equation Modeling A Multidisciplinary Journal.

[51] Parent N, Bond T, Wu A, Shapka J: Predicting patterns of problematic smartphone use among university students: a latent class analysis. Human Behav Emerg Technol 2022, 2022.

[52] Błachnio A, Przepiórka A, Gorbaniuk O, Benvenuti M, Ciobanu AM, Senol-Durak E, Durak M, Giannakos MN, Mazzoni E, Pappas IO (2019). Cultural correlates of internet addiction. Cyberpsychol Behav Soc Netw.

[53] Seung-Yup L, Donghwan L, Rong NC, Yea KD, Sera P, Jun-Gun K, Yong-Sil K, Youngjo L, Jin KD, Jung-Seok C (2018). Distinct patterns of internet and smartphone-related problems among adolescents by gender: latent class analysis. J Behav Addict.

[54] Park WK (2014). An exploitative study on college students' addiction: using psychological variables as predictors. Soc Psychol Res.

[55] Chen B, Liu F, Ding S, Ying X, Wang L, Wen Y (2017). Gender differences in factors associated with smartphone addiction: a cross-sectional study among medical college students. Bmc Psychiatry.

[56] Hakoama M, Hakoyama S (2011). The impact of cell phone use on social networking and development among college students. Am Association Behav Soc Sci J.

[57] Roberts JA, Yaya LH, Manolis C (2014). The invisible addiction: cell-phone activities and addiction among male and female college students. J Behav Addict.

[58] Geser H. Are girls (even) more addicted? Some gender patterns of cell phone usage. Sociology in Switzerland: Sociology of the Mobile Phone. 2006.

[59] Nolen-Hoeksema S (2001). Gender Differences in Depression. Curr Dir Psychol Sci.

[60] Wolniewicz CA, Rozgonjuk D, Elhai JD (2020). Boredom proneness and fear of missing out mediate relations between depression and anxiety with problematic smartphone use. Human Behav Emerging Technol.

[61] Guo N, Luk TT, Ho SY, Lee JJ, Shen C, Oliffe J, Chan SS, Lam TH, Wang MP (2020). Problematic smartphone use and mental health in Chinese adults: a population-based study. Int J Environ Res Public Health.

[62] Li JB, Mo PKH, Lau JTF, Su XF, Zhang X, Wu AMS, Mai JC, Chen YX (2018). Online social networking addiction and depression: the results from a large-scale prospective cohort study in Chinese adolescents. J Behav Addict.

[63] Liu S, Wing YK, Hao Y, Li W, Zhang J, Zhang B (2019). The associations of long-time mobile phone use with sleep disturbances and mental distress in technical college students: a prospective cohort study. Sleep.

[64] Priel B, Shamai D (1995). Attachment style and perceived social support: effects on affect regulation. Personality Individual Differences.

[65] Adams SK, Kisler TS (2013). Sleep quality as a mediator between technology-related sleep quality, depression, and anxiety. Cyberpsychology Behav Soc Networking.

[66] Cheng Y, Meng J (2021). The association between depression and problematic smartphone behaviors through smartphone use in a clinical sample. Human Behav Emerg Technol.

[67] Deursen A, Bolle CL, Hegner SM, Kommers P (2015). Modeling habitual and addictive smartphone behavior: the role of smartphone usage types, emotional intelligence, social stress, self-regulation, age, and gender. Comput Hum Behav.

[68] Kim JH, Seo M, David P (2015). Alleviating depression only to become problematic mobile phone users: can face-to-face communication be the antidote?. Comput Hum Behav.

[69] Contractor AA, Weiss NH, Elhai JD (2018). Examination of the relation between PTSD symptoms, smartphone feature uses, and problematic smartphone use. Soc Sci Comput Rev.

[70] Mei S, Chai J, Wang SB, Ng CH, Ungvari GS, Xiang YT (2018). Mobile phone dependence, social support and impulsivity in Chinese University students. Int J Environ Res Public Health.

[71] Kuss DJ, Griffiths MD (2011). Excessive online social networking: can adolescents become addicted to Facebook?. Education Health.

[72] Joiner R, Gavin J, Brosnan M, Cromby J, Gregory H, Guiller J, Maras P, Moon A (2012). Gender, internet experience, internet identification, and internet anxiety: a ten-year Followup. Cyberpsychology Behav Soc Networking.

[73] Brand M, Young KS, Laier C, Wölfling K, Potenza MN (2016). Integrating psychological and neurobiological considerations regarding the development and maintenance of specific internet-use disorders: an interaction of person-affect-cognition-execution (I-PACE) model. Neurosci Biobehav Rev.

[74] Malinauskas R, Malinauskiene V (2019). A meta-analysis of psychological interventions for internet/smartphone addiction among adolescents. J Behav Addict.

