# Supplementary material for: Distinct patterns of problematic smartphone use and related factors in Chinese college students
Source: BMC Psychiatry. 2022 Nov 30;22:747. doi: 10.1186/s12888-022-04395-z (PMC9710163; doi:10.1186/s12888-022-04395-z)
Supplement: Supplementary file 2 — Additional file 2. [file 12888_2022_4395_MOESM2_ESM.doc]

*Note*:

Grade: 1=Freshman, 2=Sophomore, 3=Junior;

Gender: 0=Female, 1=Male;

Family Origin: 0=Rural, 1=City;

One Child Family: 0=Yes, 1=No;

M1-M17: item data of Mobile Phone Addiction Index Scale (MPAI) Scale;

CES-D: the score of Center for Epidemiologic Studies Depression Scale;

PSSS: the score of Perceived Social Support Scale;

PSS: the score of Perceived Stress Scale.
